# Supplementary figures and images for: Monitoring the autophagy-endolysosomal system using monomeric Keima-fused MAP1LC3B
Source: PLoS One. 2020 Jun 8;15(6):e0234180. doi: 10.1371/journal.pone.0234180 (PMC7279612; doi:10.1371/journal.pone.0234180)

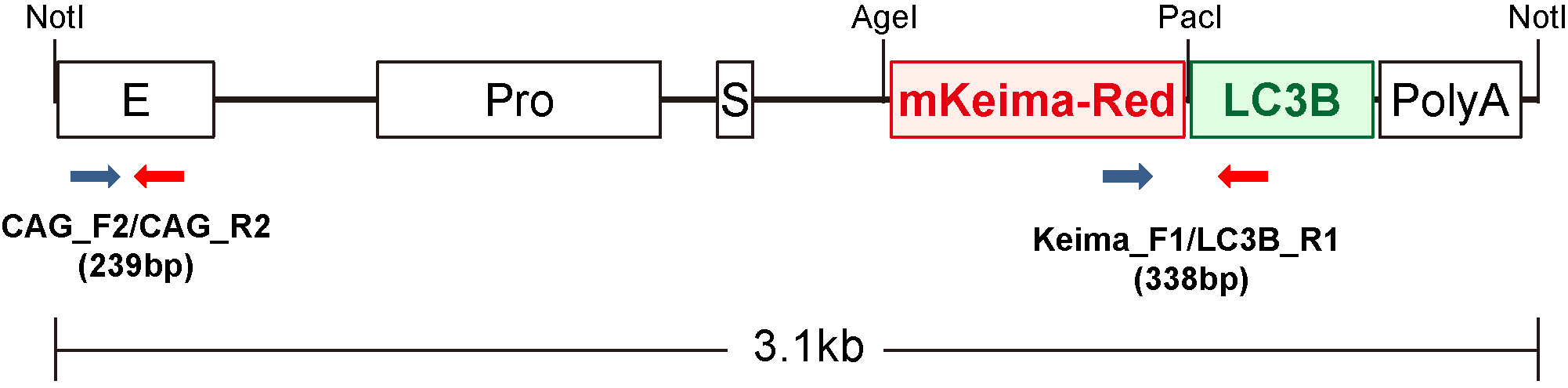

Supplement: S1 Fig — Transgene construct consists of cytomegalovirus enhancer (E), chicken β-actin promoter (Pro), rabbit β-globin splice acceptor (S), mKeima-Red cDNA, full-length human MAP1LC3B cDNA (LC3B), and rabbit β-globin poly A (Poly A). Positions of restriction enzymes used for cloning and primers for genotyping are shown. (TIF) [file pone.0234180.s001.tif]

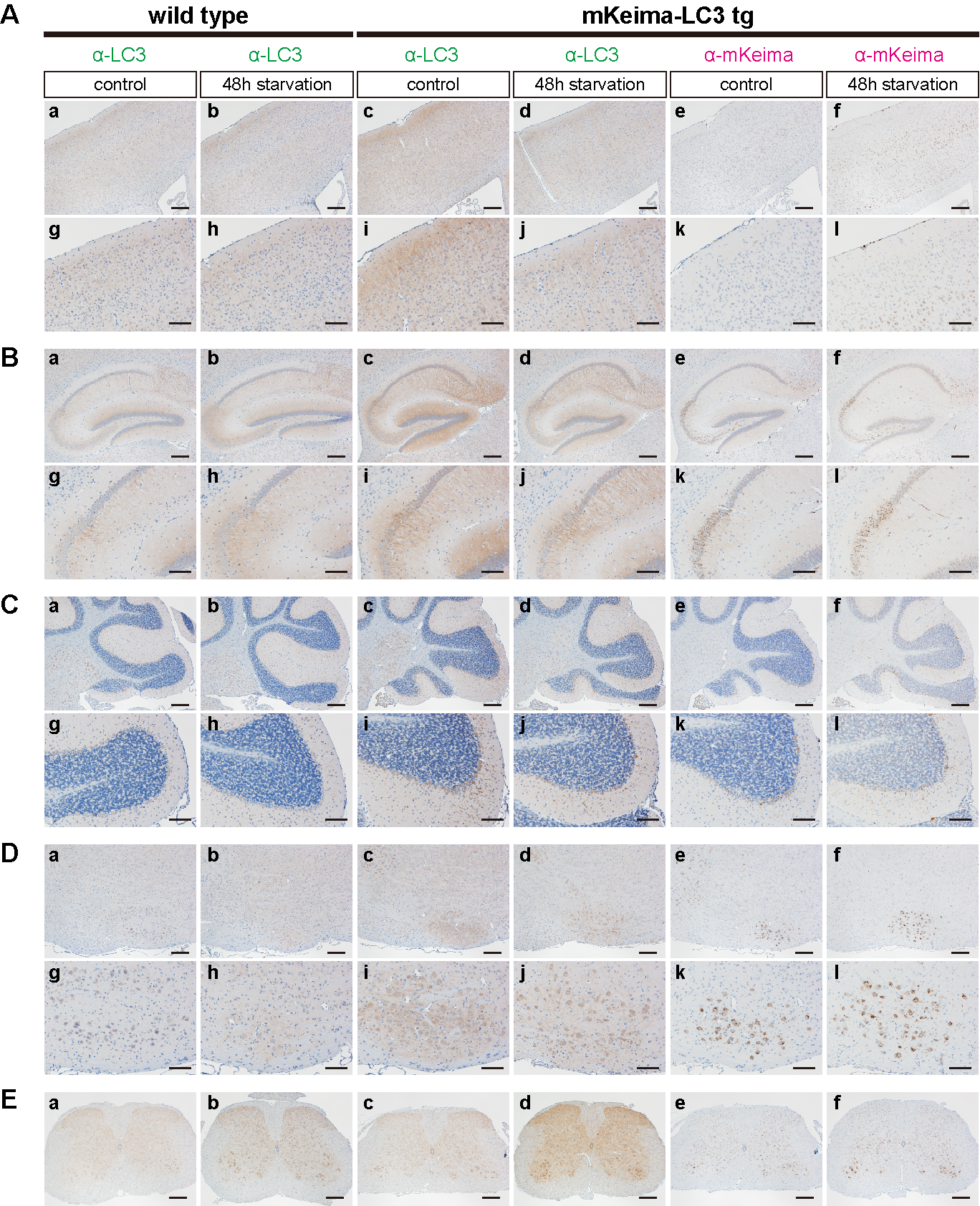

Supplement: S2 Fig — (A) Cerebral cortex, (B) hippocampus, (C) cerebellum, (D) pons (facial nucleus), and (E) lumbar spinal cord (L4-L5), which were prepared from wild-type and mKeima-LC3B transgenic (tg) mice under either fed (control) or starved (48 h) conditions, were fixed and immunostained with anti-LC3 (α-LC3) or anti-mKeima (α-mKeima) antibody. Mouse lines used in this analysis were (A-D) BDKLC3_17–1 and (E) KLC3_44. Scale bars indicate (A, a-f) 200 μm, (A, g-l) 100 μm, (B, a-f) 200 μm, (B, g-l) 100 μm, (C, a-f) 200 μm, (C, g-l) 100 μm, (D, a-f)) 200 μm, (D, g-l) 100 μm, and (E) 200 μm. (TIF) [file pone.0234180.s002.tif]

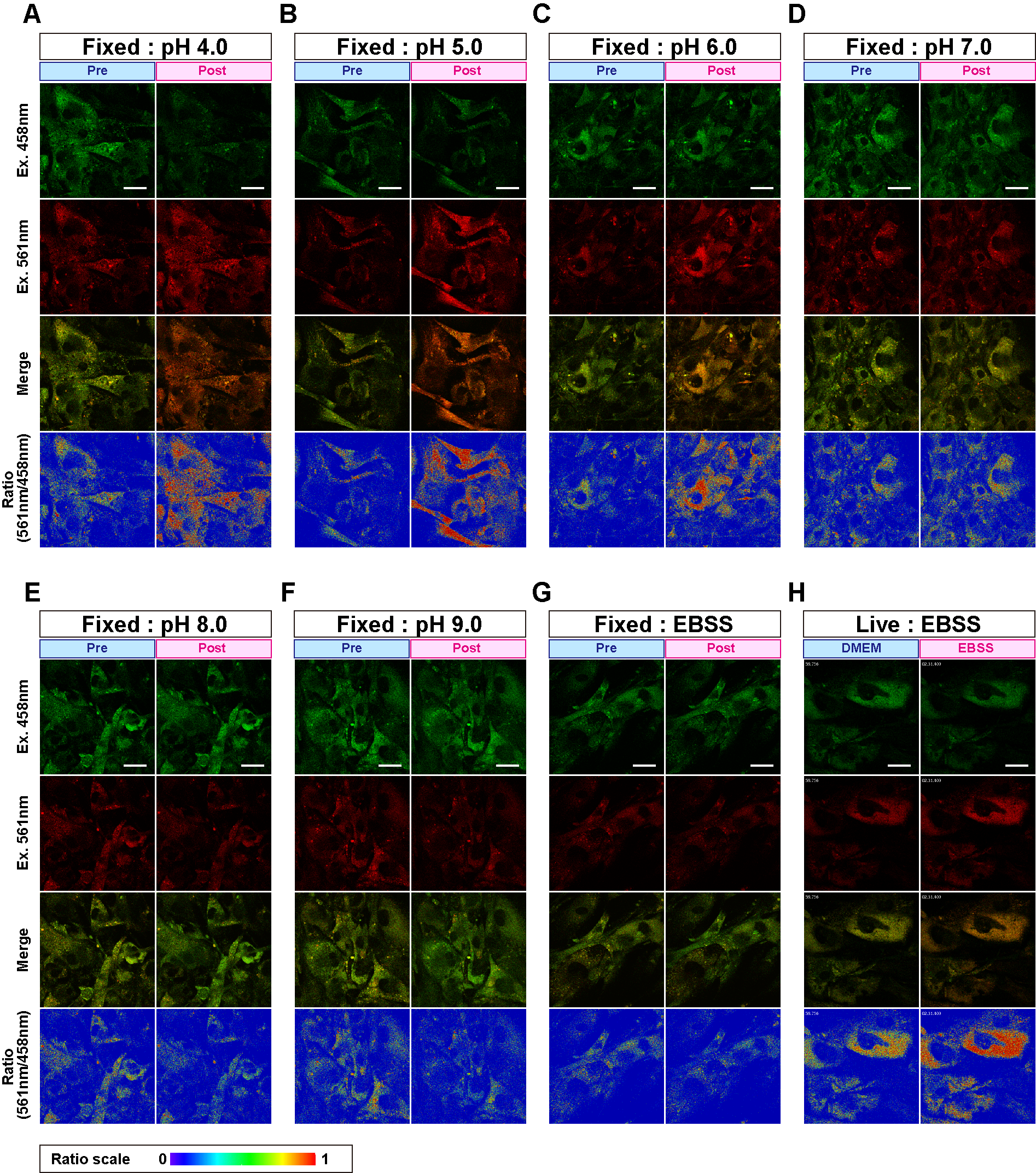

Supplement: S3 Fig — After fixation, fluorescent signals for mKeima in mKeima-LC3B-expressing MEFs, which were prepared from mKeima-LC3B-tg mice (BDKLC3_17–1), were detected (Pre). Then, fixed cells were buffered at (A) pH 4.0, (B) pH 5.0, (C) pH 6.0, (D) pH 7.0, (E) pH 8.0, or (F) pH 9.0, or treated with (G) EBSS (pH 7.4–7.6), followed by detection of fluorescent signals (Post). (H) For comparison, images for living cells treated with DMEM or EBSS are shown. Scale bars indicate 50 μm. (TIF) [file pone.0234180.s003.tif]

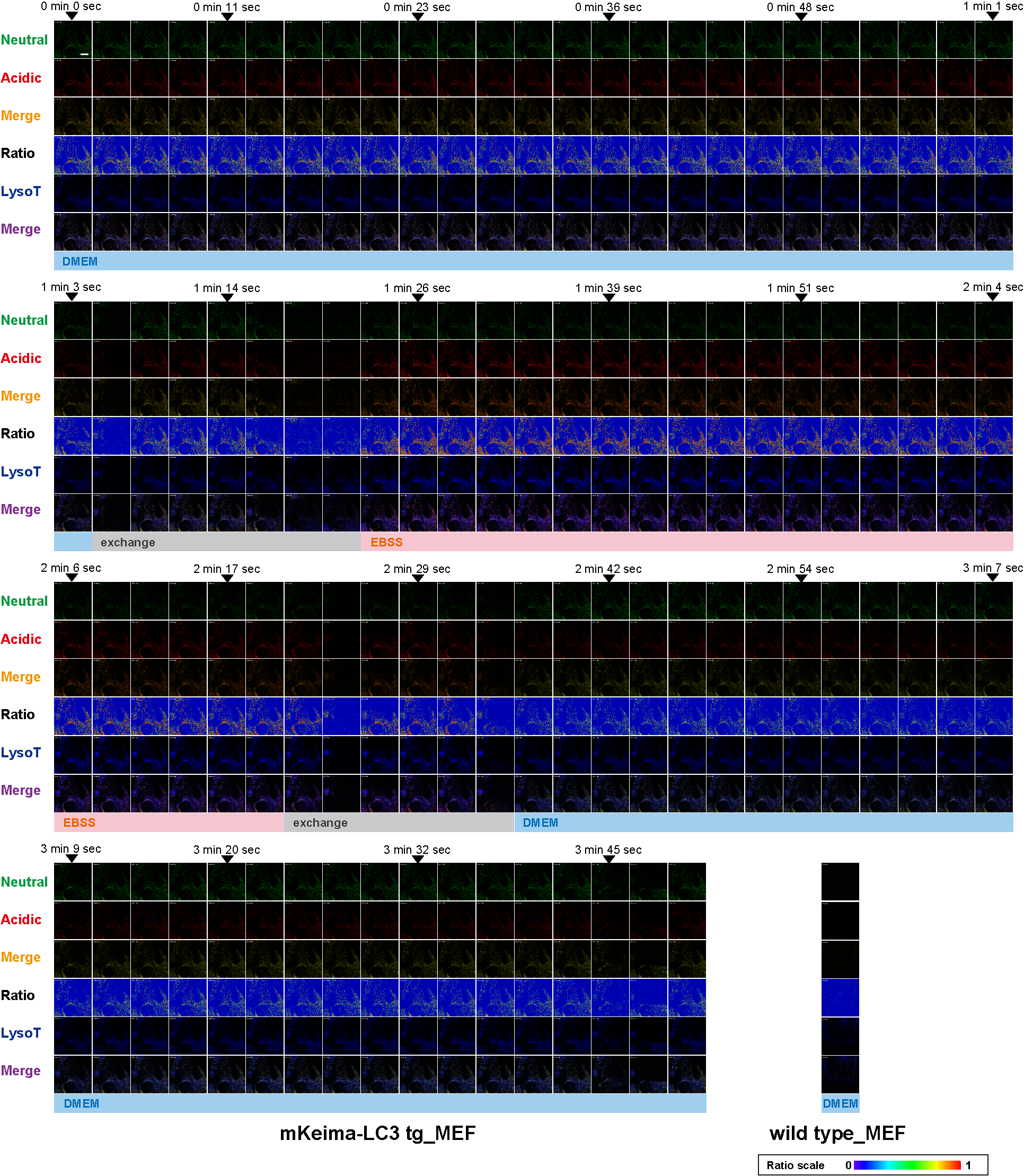

Supplement: S4 Fig — MEFs prepared from mKeima-LC3B tg mice (BDKLC3_17–1) were cultured in DMEM. After incubation in DMEM for 1 min, medium was repeatedly changed from DMEM to EBSS and from EBSS to DMEM every 80 sec. Images (Z-stack = 1) were captured every 2.5 sec. Images in each row represent as follows: upper row; Neutral (mKeima; ex. 458 nm, green), 2nd row; Acidic (mKeima; ex. 561 nm, red), 3rd row; Merge (mKeima; neutral + acidic), 4th row; ratio [mKeima; 561 nm (acidic)/458 nm (neutral)], 5th row; LysoT (LysoTracker blue; ex. 405 nm, blue), and lower row; Merge [mKeima (neutral + acidic) + LysoTracker blue]. Lap-times and medium conditions are shown in the top and bottom, respectively. As a negative control, images of wild-type MEFs cultured in DMEM are also shown. Scale bar indicates 20 μm. (TIF) [file pone.0234180.s004.tif]

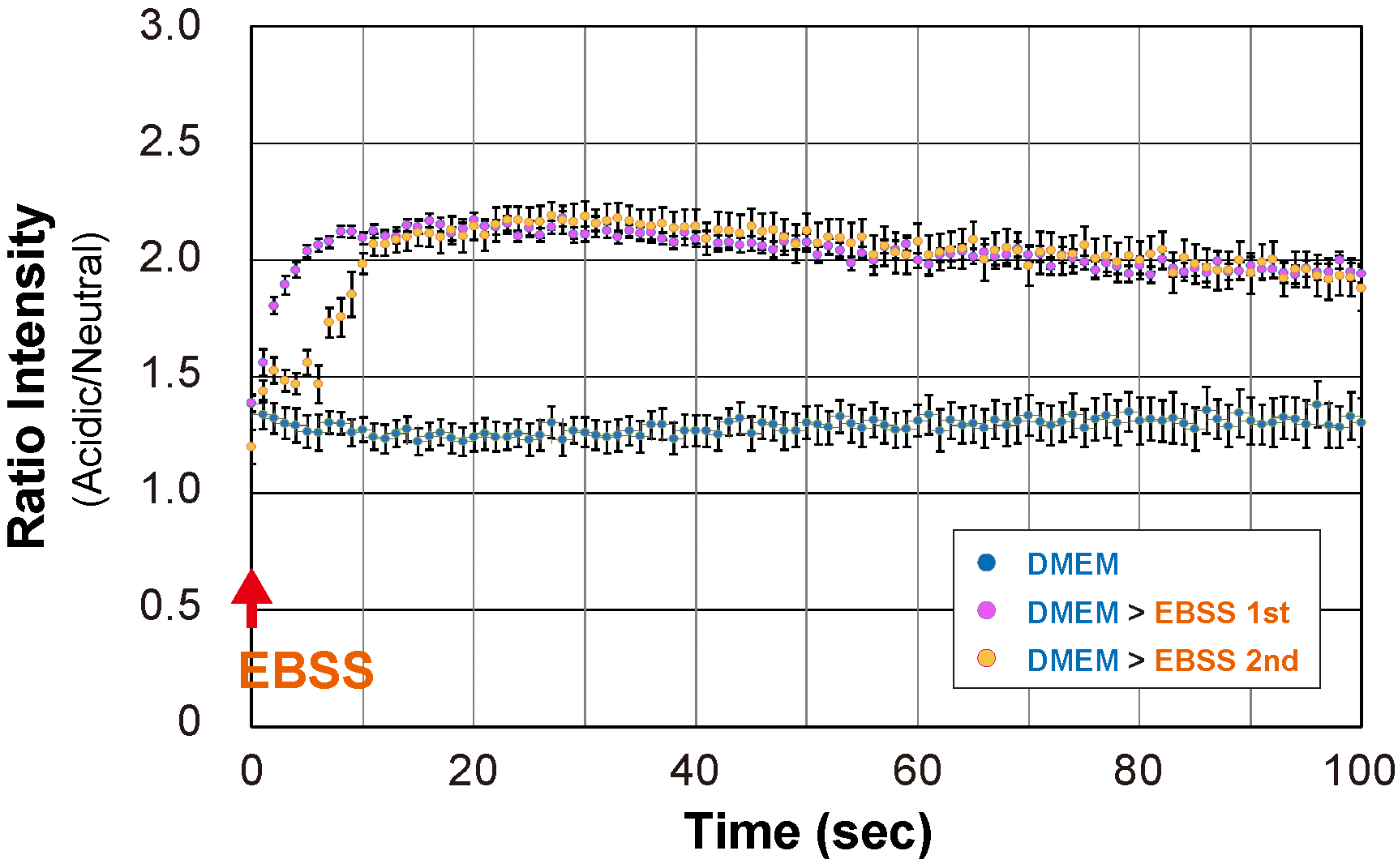

Supplement: S5 Fig — Changes in mKeima-derived fluorescent signal ratio (Acidic/Neutral) under transitional conditions from nutrient-rich (DMEM) to starvation (EBSS) states are shown. Two independent experiments, in which data were captured for 100 sec at 1 sec intervals [EBSS 1st (magenta circle); observed field; n = 29 (total number of cells; n ≈ 350) and EBSS 2nd (yellow circle); observed field; n = 55 (total number of cells; n ≈ 670)], were performed. Signal ratio under nutrient-rich conditions [DMEM (blue circle); observed field; n = 50 (total number of cells; n ≈ 610)] was also monitored as a control. Values are expressed as mean ± (S.E.M.). (TIF) [file pone.0234180.s005.tif]

**Fig 1A**

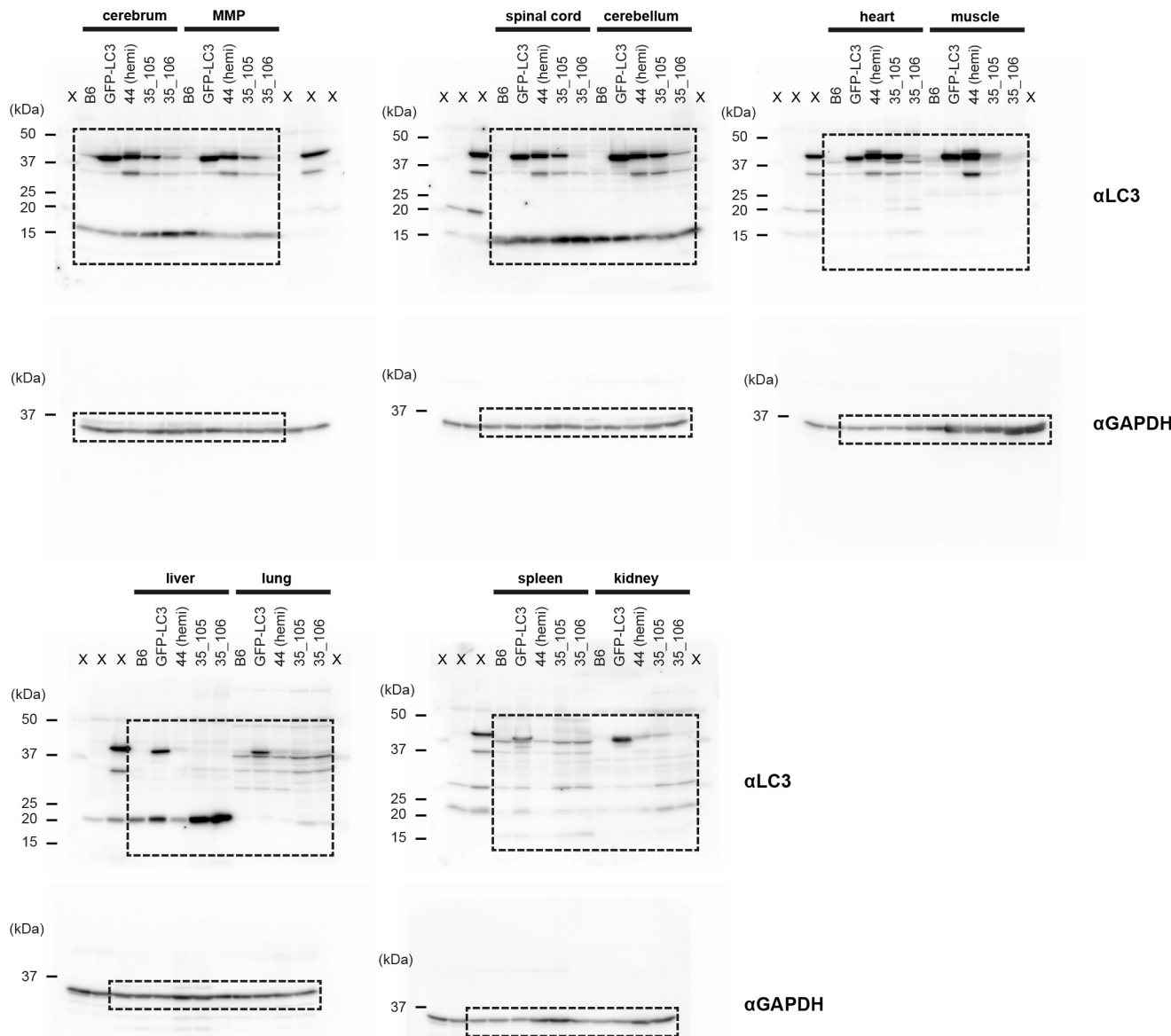

**Fig 1B**

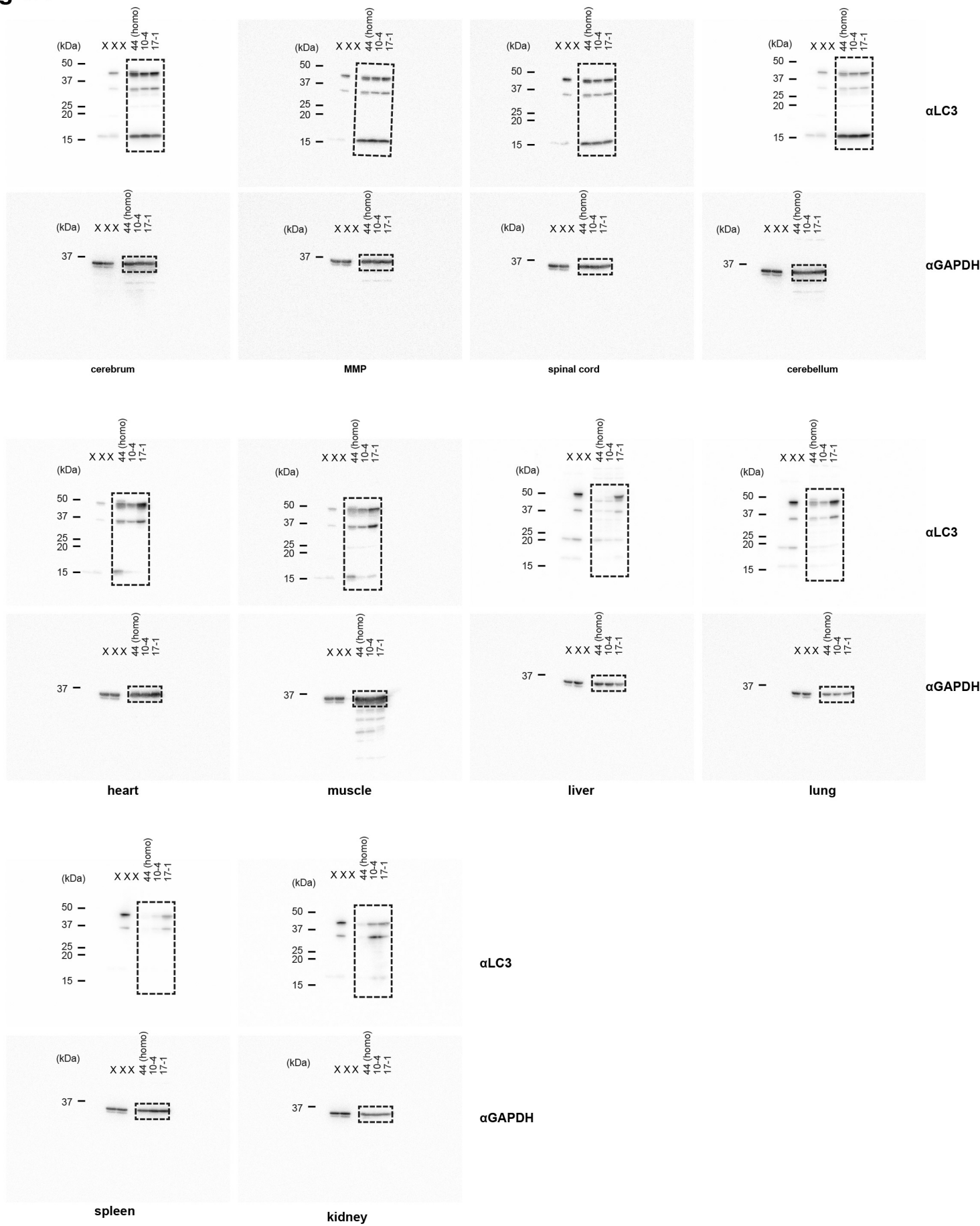

**Fig 2**

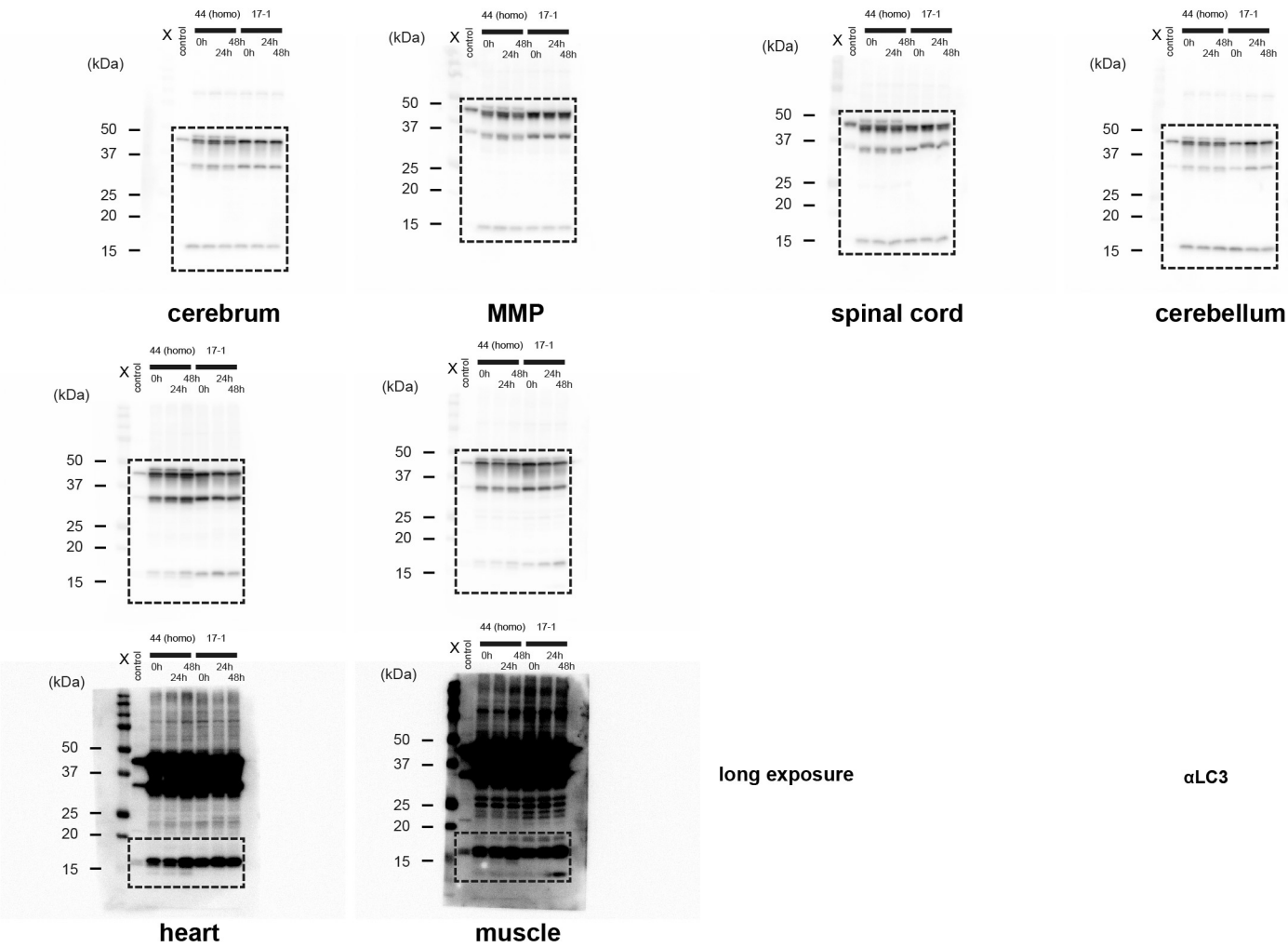

**Fig 4A**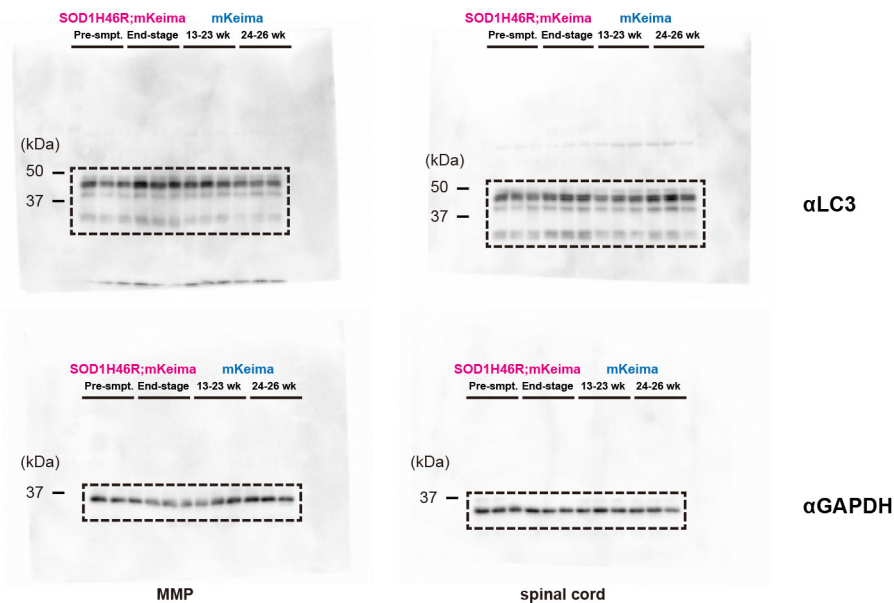**Fig 4B**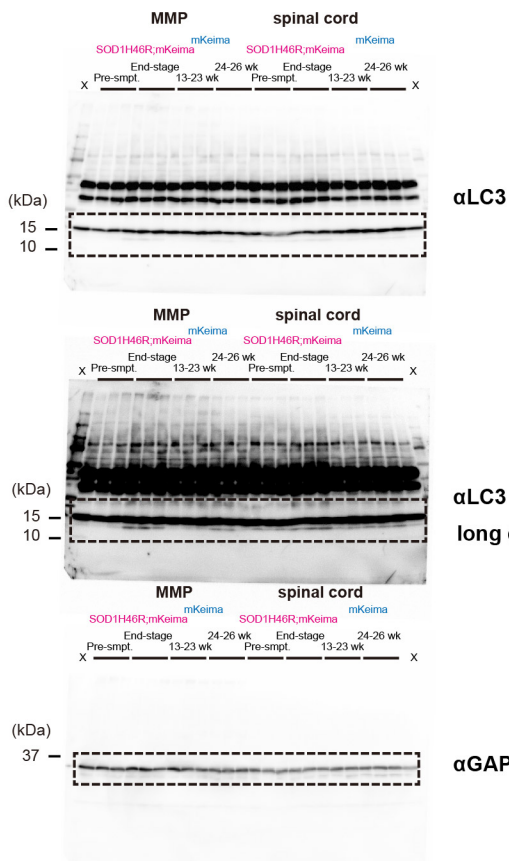**Fig 4C**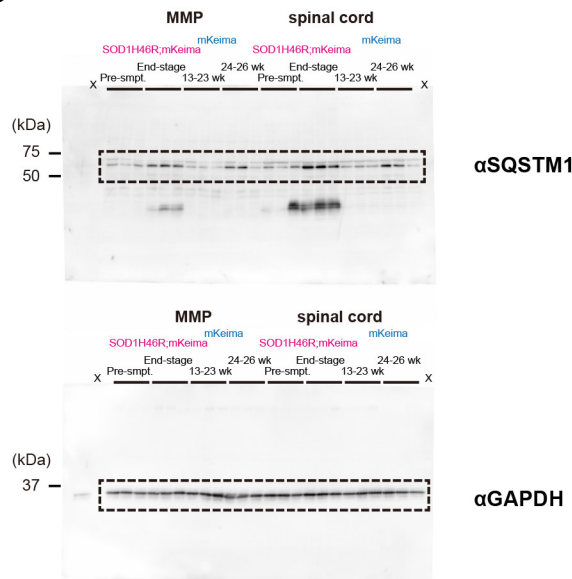

# Fig 6A

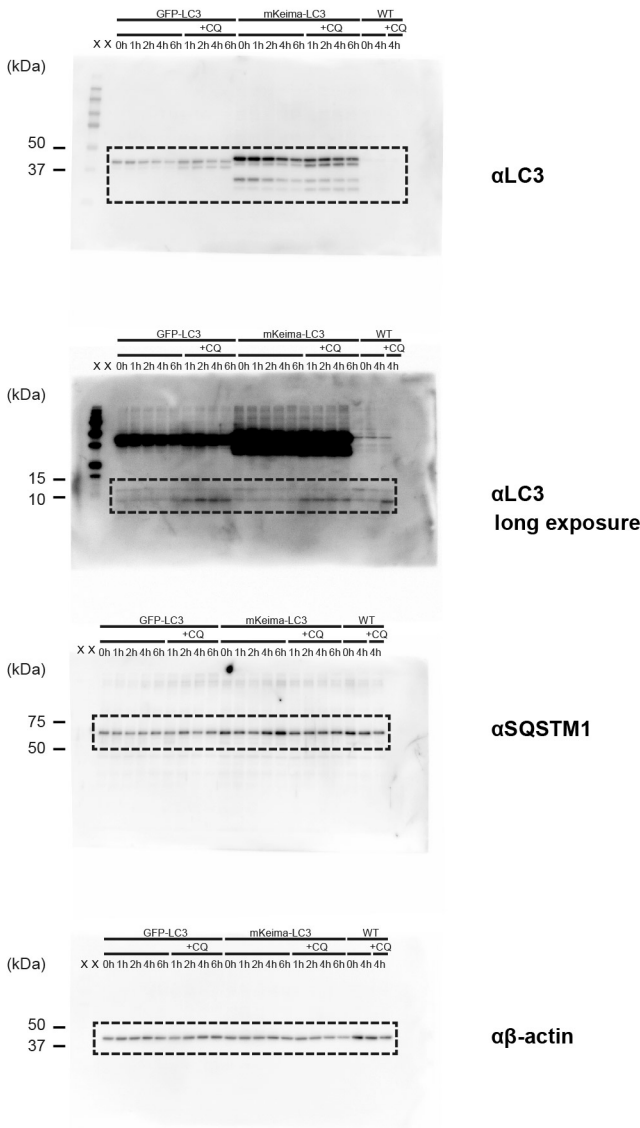

Supplement: S1 File — Immunoreactivities were visualized with Immobilon-Western Chemiluminescent HRP Substrate (Millipore) and analyzed using Ez-Capture Analyzer (ATTO). (PDF) [file pone.0234180.s022.pdf]
