# Supplementary material for: Monitoring the autophagy-endolysosomal system using monomeric Keima-fused MAP1LC3B
Source: PLoS One. 2020 Jun 8;15(6):e0234180. doi: 10.1371/journal.pone.0234180 (PMC7279612; doi:10.1371/journal.pone.0234180)
Supplement: Attachment — Submitted filename: Response_to_Reviewers.docx [file pone.0234180.s023.docx]

December 16, 2019

Dr. Vladimir Trajkovic

Academic Editor

PLOS ONE

Dear Dr. Trajkovic,

We greatly appreciate the excellent reviews. A revision of our manuscript (PONE-D-19-24727) by Hideki Hayashi et al. has been completed. Please find uploaded files for our revised manuscript entitled **“Monitoring the autophagy-endolysosomal system using monomeric Keima-fused MAP1LC3B”** by Hideki Hayashi, Ting Wang, Masayuki Tanaka, Sanae Ogiwara, Chisa Okada, Masatoshi Ito, Nahoko Fukunishi, Yumi Iida, Ayaka Nakamura, Ayumi Sasaki, Shunji Amano, Kazuhiro Yoshida, Asako Otomo, Masato Ohtsuka and Shinji Hadano. We also attached the manuscript, in which corrections that we made were highlighted as red-colored letters, as a separate file.

To answer the comments and/or questions provided by the reviewers, we have conducted an additional set of experiments, whose results are exclusively included in our revised manuscript. Comments and questionnaires from reviewers are itemized and accompanied with our answers/responses as bellows.

**Reviewer #1’s comments:**

*1. The authors analyzed pH sensitivity of Keima LC3 after fixation. it is better to perform this before fixation.*

**- Response to this comment:**

We appreciate the reviewer’s important comment. As suggested by the reviewer, monitoring the pH sensitivity without fixation must be an ideal way to do. However, without fixation, which means the cells still alive, the incubation with acid or alkaline buffer solutions, in particular under extreme pH conditions, is harmful to cells; sometimes resulting in cell death. Under such conditions, analysis of pH sensitivity of expressed mKeima-LC3B may be extremely difficult. In addition, even after fixation, mKeima seems to keep the variable excitation spectra in a pH-dependent fashion. Thus, we are currently believing that the fixation of cells is the best way to stably measure and estimate the pH sensitivity of mKeima-LC3B molecules *in situ*. Alternatively, it may also be possible to biochemically measure the sensitivities using purified proteins. Indeed, the pH sensitivity of mKeima using purified protein has already been reported in the original publication (Katayama et al, Chem Biol 18: 1042, 2011). However, in our hand, purification of the mKeima-LC3B fusion protein has been unsuccessful thus far. These are the reasons why we have currently adopted the fixed cells in this study.

*2. The study reveals rapid and reversible autonomous change of pH in LC3-positive autophagosomes before the vesicle-fusion. This is an interesting observation needs further validation. The methodology is having potential application in diverse areas of biology including drug screening.*

**- Response to this comment:**

We appreciate the reviewer’s invaluable comments. As pointed out by the reviewer, we have not shown the direct evidence whether a rapid and reversible change of pH in mKeim-LC3B-positive autophagosomes occurs autonomously without committing the vesicle-fusion. Since we were unable to clearly distinguish between nascent/immature and maturated mKeima-LC3B-positive autophagosomes by staining with LysoTracker (see Fig 7), we tried to do an additional set of experiments of a live-cell imaging. Although the pharmacological inhibition of vesicular proton pumps by chloroquine (CQ) resulted in suppression of nutritional-condition-dependent rapid changes in vesicular pH (see S21 movie); yet such suppression was still reversible (see Fig 9F), we had been unable to show the commitment of vesicle fusion by this procedure. Thus, due to lack of sufficient evidences, we decided to decline the over-stated sentences that were describing “the commitment of vesicle fusion” in the phenomena of a rapid and reversible pH changes, in order to avoid the confusion as well as misunderstanding by the readers.

**Reviewer #2’s comments:**

*1. It is not clear to me what the added value is of adding LC3 to the existing mKeima probe. mKeima is a well-validated autophagy probe that is taken up into autophagosomes after autophagy induction and allows distinction of autophagosomes from lysosomes. What is the advantage of attaching LC3 to mKeima? The rationale for making this fusion construct should be explained much better.*

**- Response to this comment:**

We appreciate the reviewer’s comments. Generally, macroautophagy (autophagy hereafter) is implicated in two different types of degradation; non-selective and selective degradation. Recent evidences have uncovered that dysregulation of the selective cargo-degradation system rather than non-selective one is associated with many pathological conditions such as cancer, inflammation and neurodegenerative diseases, and that LC3 and other autophagy receptor molecules containing the LIR (LC3-interacting region), such as SQSTM1/p62, NBR1 and optineurin, contributes to such disorders. Since LC3 is known to not only directly bind to autophagosomal membranes, but also simultaneously to several autophagy receptors, attaching LC3 molecule to mKeima has a strong advantage to monitor the selective-degradation activity, as has already been shown by many previous publications using GFP-LC3 fusion molecules. By contrast, in the original paper by Katayama et al (Chem Biol 2011), lone mKeima can become a probe for non-selective autophagy but not for selective ones. In this revised manuscript, we added the phrase “the selective cargo-degradation system” in the section of Introduction, to facilitate the proper understanding by the readers.

*2. It is very strange that mKeima-containing acidic vesicles (confirmed to be lysosomes by containing with LysoTracker) become neutral within 10 seconds after switching from EBSS to DMEM. It seems very unlikely that the proton gradient across the lysosomal membrane can be completely abolished within 10 seconds after this medium switch. Which signaling pathway could possibly explain such rapid de-acidification? This finding seems to hint at some possible artifact related to the medium switch. More data would be required to make this finding more convincing.*

**- Response to this comment:**

We appreciate the reviewer’s invaluable comments. First, as already mentioned in response to the Reviewer#1, we were unable to clearly distinguish between nascent/immature and maturated mKeima-LC3B-positive autophagosomes by staining with LysoTracker (Fig 7). It was also noted that LysoTracker signals were not changed by the DMEM-EBSS medium switch (revised S9 Movie). We are currently speculating that LysoTracker-labeled vesicles represent not only fully matured autolysosomes/lysosomes but also immature autophagosomes/late endosomes as well as amphisomes, which are emerged by a gradual acidification of autophagosomes/endosomes through the activation of vesicular proton pumps.

To further response to the reviewer’s questions, we have conducted an additional set of experiments. In order to test whether the medium change itself affected fluorescent signals, we performed a live-cell imaging under repeated changes of the same medium; i.e., DMEM to DMEM, or EBSS to EBSS. Although some signal fluctuations during the medium exchange were observed; which might be due to the deflection of laser-beam by transiently lowering level of medium solution, there were no observable differences in the acid/neutral signal ratio between before and after changing the medium (revised S4 Movie and S5 Movie). Therefore, medium change process itself may not affect the acidic/neutral signal ratio of mKeima-LC3B-positive compartments. Next, to test whether a rapid and reversible change in intraluminal pH of mKeima-LC3B-positive vesicles was associated with genuine maturation of autophagosomes and/or endosomes, we conducted a live-cell imaging as well as quantification of acidic/neutral signals for mKeima-LC3B in the presence or absence of CQ. All the results obtained are exclusively described in a separate paragraph of the Results section and related additional figures and movies were included (Fig 9, S15-S21 Movies). Although the deacidification by CQ proceeded much slower than did by medium switch from EBSS to DMEM, the pharmacological inhibition of vesicular proton pumps by CQ results in suppression of nutritional-condition-dependent rapid changes in vesicular pH (revised S21 movie). Importantly, such suppression was still reversible (revised Fig 9F). The results indicate that a rapidly changeable intraluminal pH of mKeima-LC3B-positive vesicles depends, at least in part, on the process of the authentic maturation system of autophagosomes and/or endosomes.

Based on our results, it is possible that continuous and persistent activation of proton pumps may be required to keep autophagosomes/endosomes towards acidic in their reversible phase. Under nutrition-rich conditions, the proton pump activity may rapidly decline via a certain signaling pathway. Consequently, the ability of maintaining an intraluminal acidity is rapidly lost, resulting in autophagosomes with neutral pH within 10 sec. Conversely, when starved, massive activation of proton pumps may explain a rapid acidification of autophagosomes. Thus far, we could not fully explain the mechanism and/or signaling pathway for these rapid-reversible phenomena in an evidence-based manner. Further studies are warranted as stated in the manuscript.

*3. The in vivo data in the transgenic mice are not adding much and could be removed from the manuscript*

**- Response to this comment:**

We appreciate the reviewer’s important suggestions. As pointed out by the reviewer, the results were rather negative indeed. However, if allowed, we would like to show those data in order to notify the negative side of mKeima molecule in the use of *in vivo* experiments to the readers.

*4. What is the meaning of the mKeima-LC3’ (as opposed to mKeima-LC3) band in the western blot figures?*

**- Response to this comment:**

We appreciate the reviewer’s comments. To avoid the confusion, we clearly added the definition of “mKeima-LC3’” in the legends of Fig 1, Fig 2, Fig 4 and Fig 6.

We hope that all the changes we made meet your acceptance of the manuscript for publication in **PLoS ONE**. Thank you for your generous consideration. We are looking forward to hearing from you soon.

Sincerely yours,

Shinji Hadano, Ph.D.
---------------------------------------------------
Professor of Department of Molecular Life Sciences

Tokai University School of Medicine

143 Shimokasuya, Isehara, Kanagawa 259-1193, JAPAN

TEL: +81-463-93-1121 (ext. 2567)

FAX: +81-463-93-3965

E-mail: shinji@is.icc.u-tokai.ac.jp
