# Supplementary material for: Monitoring the autophagy-endolysosomal system using monomeric Keima-fused MAP1LC3B
Source: PLoS One. 2020 Jun 8;15(6):e0234180. doi: 10.1371/journal.pone.0234180 (PMC7279612; doi:10.1371/journal.pone.0234180)
Supplement: Attachment — Submitted filename: Response_letter_R2.docx [file pone.0234180.s024.docx]

March 9, 2020

Dr. Vladimir Trajkovic

Academic Editor

PLOS ONE

Dear Dr. Trajkovic,

We greatly appreciate the excellent reviews. A revision of our manuscript (PONE-D-19-24727R1) by Hideki Hayashi et al. has been completed. Please find uploaded files for our revised manuscript entitled **“Monitoring the autophagy-endolysosomal system using monomeric Keima-fused MAP1LC3B”** by Hideki Hayashi, Ting Wang, Masayuki Tanaka, Sanae Ogiwara, Chisa Okada, Masatoshi Ito, Nahoko Fukunishi, Yumi Iida, Ayaka Nakamura, Ayumi Sasaki, Shunji Amano, Kazuhiro Yoshida, Asako Otomo, Masato Ohtsuka and Shinji Hadano. We also attached the manuscript, in which corrections that we made were highlighted as red-colored letters, as a separate file.

To answer the comments and/or questions provided by the reviewer#2, we have tried to add more detailed explanations with additional citations mainly to the sections of “Introduction” and “Discussion”, and at the same time, to refine them as precisely as possible. Comments and questionnaires from reviewers are itemized and accompanied with our answers/responses as bellows.

**Reviewer #2’s comments:**

*1. It remains unclear what the added value is of adding LC3 to the existing mKeima probe. The response of the authors to this comment does not make sense. LC3 is involved in selective as well as non-selective autophagy. The response by the authors that “attaching LC3 molecule to mKeima has a strong advantage to monitor the selective degradation activity” is simply not correct.*

**- Response to this comment:**

We appreciate the reviewer’s comment. We would like to admit our misunderstanding and totally agree with what the reviewer has pointed-out; i.e., LC3 is involved both in selective and non-selective autophagy. To facilitate the readers’ proper understanding of our aim as well as reason as to why the LC3 molecule attached to mKeima, we tried to correct and explain them by adding several sentences to the section of “Introduction” (see lines 68-77 in the correction-highlighted manuscript).

*2. The finding that mKeima-containing acidic vesicles become neutral within 10 seconds after switching from EBSS to DMEM remains very strange and the mechanism remains completely unexplained.*

**- Response to this comment:**

We appreciate the reviewer’s invaluable comments. As the reviewer has pointed out, a phenomenon of the rapid deacidification (~ within 10 sec) of mKeima-LC3B-resided compartments, which is induced by changing the media from EBSS to DMEM, is also totally unexpected to us. Thus far, to the best of our knowledge, there have been no studies demonstrating the rapid deacidification of autophagosomes and/or endocytic vesicles, let alone the mechanisms of such phenomena. Although data showing such rapid deacidification are reproducibly obtained, at least, by our hand, we honestly say that we do not have enough abilities to clarify their molecular mechanisms as well as physiological significances in an experimental evidence-based manner within a designated period of this revision time. Therefore, in this revision, we decided to add more detailed explanations in the section of “Discussion”, and proposed the hypothetical mechanisms that were drawn from the current findings on the acidification and deacidification of endocytic vesicles in conjunction with the regulation of the V-ATPase functions under amino acid deplete as well as replete conditions (see lines 619-670 in the correction-highlighted manuscript). Further, we added a separated paragraph to the section of “Discussion” to explain in more detail on arising issues on LC3-labeled compartments (see lines 671-680 in the correction-highlighted manuscript).

By submitting the manuscript to **PLoS ONE**, we understand that “the work described has not been submitted for publication, in whole or in part, elsewhere and all the authors listed have approved the manuscript that is enclosed”. We understand that “should the submitted material be accepted for publication in PLoS ONE, we will automatically transfer the copyright to the publisher”. Further, the authors have declared that no competing interests exist.

We hope that all the changes we made meet your acceptance of the manuscript for publication in **PLoS ONE**. Thank you for your generous consideration. We are looking forward to hearing from you soon.

Sincerely yours,

Shinji Hadano, Ph.D.
---------------------------------------------------
Professor of Department of Molecular Life Sciences

Tokai University School of Medicine

143 Shimokasuya, Isehara, Kanagawa 259-1193, JAPAN

TEL: +81-463-93-1121 (ext. 2567)

FAX: +81-463-93-3965

E-mail: shinji@is.icc.u-tokai.ac.jp
